# Child and youth mental health and wellbeing before and after returning to in-person learning in secondary schools in the context of COVID-19

**DOI:** 10.3389/fpubh.2023.1212297

**Published:** 2023-09-01

**Authors:** Lei Qian, Robert McWeeny, Cheryl Shinkaruk, Andrew Baxter, Bo Cao, Andy Greenshaw, Peter Silverstone, Hannah Pazderka, Yifeng Wei

**Affiliations:** ^1^Department of Psychiatry, University of Alberta, Edmonton, AB, Canada; ^2^Edmonton Catholic School Division, Edmonton, AB, Canada; ^3^Alberta Health Services Calgary Zone, Calgary, AB, Canada

**Keywords:** mental health, wellbeing, youth, stress, help seeking, COVID-19, secondary school, in person learning

## Abstract

**Background:**

As children reintegrate with in-person classroom learning after COVID-19, health and education institutions should remain mindful of students’ mental health. There is a paucity of data on changes in students’ mental health before, during and after their return to in-person classroom learning.

**Methods:**

We collected and analyzed data on self-reported wellbeing, general mental health, perceived stress, and help-seeking attitudes from grade 7–12 students in a Catholic school division in Canada (*n* = 258 at baseline; *n* = 132 at follow-up). Outcomes were compared according to demographic differences such as gender, grade level, experience accessing mental health services, and presence of support staff between baseline and follow-up. Effects of time points and each demographic variable on each outcome and on the prediction of students’ mental health were also analyzed.

**Results:**

No significant differences were apparent for outcomes between baseline and follow-up. However, specific subgroups: junior high students, male students, students who had not accessed mental health services, and students who had access to support-staff had better outcomes than their counterparts. From baseline to follow-up, male students reported mental health decline [Mean = 11.79, SD = 6.14; Mean = 16.29, SD = 7.47, *F*(1, 333) = 8.36, *p* < 0.01]; students who had not accessed mental health services demonstrated greater stress [Mean = 20.89, SD = 4.09; Mean = 22.28, SD = 2.24, *F*(1, 352) = 6.20, *p* < 0.05]; students who did not specify a binary gender reported improved general mental health [Mean = 19.87, SD = 5.89; Mean = 13.00, SD = 7.40, *F*(1, 333) = 8.70, *p* < 0.01], and students who did not have access to support-staff improved help-seeking attitudes [Mean = 22.32, SD = 4.62; Mean = 24.76, SD = 4.81; *F*(1, 346) = 5.80, *p* < 0.05]. At each time point, students indicated parents, guardians, and close friends as their most-preferred help-seeking sources. High stress predicted lower wellbeing at baseline, but higher wellbeing at follow-up.

**Conclusion:**

Students presented stable mental health. Subgroups with decreased mental health may benefit from extra mental health support through building capacity among teachers and health care professionals to support students following public health emergencies.

## 1. Introduction

COVID-19 impacted the lives of school-aged children and adolescents in multiple ways. Over the course of the pandemic, the students’ school attendance routines were disrupted by school closures, prompting a transition toward digital learning platforms and hybrid educational delivery solutions. Globally, over 1.5 billion students and youth experienced educational disruption from the pandemic (1).

Stress from lifestyle transformations due to the pandemic underlie deleterious changes in mental health and wellbeing in children and adolescents. The lack of sufficient support may increase adolescents’ likelihood of developing stress-induced anxiety symptoms and associated negative coping skills such as self-blame, denial, and substance misuse (2–4). The stress-coping theory (5, 6) has suggested how individuals cope with stress, including access to timely and appropriate resources, can impact mental health outcomes. Many social supports exert protective effects against the impacts of prolonged and chronic stress (5–8), including positive connections with families, teachers, peers, and institutions youth interact with, such as school communities. For example, there is evidence espousing protective impacts of positive parent–child communications against mental health problems in school-age students during remote-learning periods (2, 9). Additionally, students with greater pre-pandemic school and peer connectedness demonstrated better mental health and wellbeing pre-pandemic, during the first lockdown, and upon returning to in-person classroom learning (10).

The global impact of COVID-19 pandemic on children and youth varied across countries and regions (11), as demonstrated by the responses and measures under different policies. School closure is one of the important measures of social distancing to deal with the pandemic challenges (12). Recent research compared how 10 countries, including Canada, responded to the pandemic in its initial 2 years (13). The results suggested that in Canada the duration of primary and secondary school closures was 51 weeks, the second longest among the countries compared. Canada was also among the strictest countries in implementing and sustaining stringent measures of social distancing and travel restrictions. Other countries, such as Uganda and Bolivia, were reported to close schools for almost 2 years during the pandemic (11). As we know more about the virus and disease and how to mitigate the disease transmission, “reopening schools should be countries’ highest priority,” as suggested by a joint UNESCO, UNICEF, and World Bank report (14). The current study was designed to explore student mental health and inform decision-making on how to support students transitioning back to in-person classroom learning.

According to a recent systematic review (15), school-implemented pandemic response measures fall under 4 broad categories, including measures to reduce contact, measures to make contact safer, surveillance and response measures (e.g., testing and isolation, and symptomatic screening and isolation), and multicomponent measures that employed several response measures in different categories. These measures had positive effects, such as the reduction in transmission and hospitalization, but also decreased the number of days spent in schools. This review found that studies investigating the impact of these measures demonstrated low or very low quality, which was influenced by the heterogeneity of studies design (e.g., the mix of modeling studies, observational studies, and quasi-experimental studies, making cross-study comparison impossible) and the inconsistency of outcome measures. Therefore, these results presented challenges to interpret in the context of student mental health and warranted the need to investigate student mental health when they were back to in-person classroom learning.

Earlier COVID-19 research demonstrated some common mental health problems and mental disorders among children and youth such as anxiety, depression, post-traumatic stress disorder, and substance use disorder (16–20). There is no general consensus of overall global data on prevalence of mental health problems and disorders during the pandemic. This is illustrated by reports of a substantial range in prevalence of anxiety symptoms (1.8–49.5%), depression symptoms (2.2–63.8%), anger (30.0–51.3%), and irritability (16.7–73.2%) (9). In 2020, Stats-Canada compared mental health survey responses from children and youth before the pandemic in 2018, and during the pandemic in 2020. Overall, self-perceived mental health declined between 2018 and 2020 in all age-groups, but most-substantially among those aged 15–24 (21). Children and adolescents were also impacted by social and economic pandemic effects such as altered routines, disrupted sleep, disrupted family income, food insecurity, and domestic issues (22, 23). Lack of access to health and community services pose significant barriers for individuals struggling with mental health concerns and impede schools’ ability to respond effectively to student mental health challenges and promote student mental health (20, 24).

By contrast, a Canadian national survey of youth ages 10–17 indicated that significant portions of children and youth reported thoughts and concerns during the pandemic (25), rather than mental health problems and mental disorders among children and youth that were reported above. In this survey, many respondents felt bored (71%), quite normal (41%), missed their friends (54%), were academically unmotivated (60%), and were generally disliking their current social isolation (57%). That survey further found that students’ biggest concerns included continuing remote learning the next year (29%) and on this current school year (27%). Most students already engaged in remote learning felt that they were keeping up with schoolwork (75%). Another Canadian study focused on youth ages 12–18 upon schools reopening and found that students’ stress reactions, problematic conduct, negative affect, and attention concerns were all below critical thresholds (26). Severity of mental health problems and disorders among students appears to vary demographically. For example, senior high school students and female students had greater depression and anxiety symptoms than junior high students or male students (26–28).

Previous school-based studies in western Canada provided evidence about how resilience improved depression and suicidality over time in high-risk adolescents (29, 30). Researchers employed a multimodal resilience-building program in youth struggling with depression and suicidality. The 12-weeks post-test results demonstrated a 15% reduction in depression scores across all-groups, 10% reduction in anxiety, and 61% of students who were actively suicidal at baseline no-longer qualified as “at-risk” (29). After 15-months follow-up, reductions in depression and suicidality remained statistically significant compared to baseline. These findings support the reasoning that ameliorating diverse psychosocial problems during and following periods of duress may provide significant benefit to youth mental health outcomes (30).

As different parts of the world exercised varied pandemic recovery strategies, it is important to remain mindful of unknown impacts on youth mental health as they reacclimate to in-person classroom learning. Prevalence of symptoms of mental health problems and disorders during the COVID-19 pandemic may vary across time-domains. For example, a 2021 study reported rising prevalence of depression and anxiety symptoms during the course of the pandemic (28). There is an increasing body of international findings about student mental health amidst recurring school closures and reopening during the pandemic. For example, students ages 13–14 in the United Kingdom experienced decreased anxiety during the first lockdown compared to pre-pandemic; and increased anxiety upon returning to school (10). Chinese students ages 9–16 who undertook mental health assessments during pandemic outbreak, school reopening (5 months post-outbreak), and 1 month after school reopening exhibited increased depression and suicidality indicators after school reopening (31). Another survey conducted at school reopening (32), compared mental health of youth who had returned to school for 2 weeks to 2 months and those who were homeschooling. Students who returned to school had more adverse behaviors, higher rates of parent–child conflict, prolonged homework time, increased sedentary time, and sleep problems than students who were schooling at home. In a correlational analysis (33), adolescents who self-reported a better relationship with their parents during distance-learning demonstrated lower depression and anger scores after returning to school for 3 months, compared to student counterparts with worse self-reported relationships with parents.

As students consistently re-enter classrooms following pandemic-related cycles of school-closures in Canada, there is limited research considering youth mental health and wellbeing at all stages of pandemic response and recovery in Canada. It is essential that researchers and schools gain access to relevant data to inform how to optimize school-experience, support, and expectations for students reacclimating to in-person learning and best supporting their mental health. The present study addresses this gap and serves to inform education and health institutions in facilitating effective transitions to in-person learning while considering the ambiguous context of post-pandemic mental health in youth. This study was approved by the ethics committee of the authors institution (#Pro00112061).

## 2. Methods

### 2.1. Participants

A cross sectional survey design was used to explore youth mental health upon students’ indefinite return to in-person learning and at 3-months follow-up. There were no previous population studies to evaluate the outcomes in the present study. Therefore, for sample size estimation, we followed an intervention protocol (34) measuring the same outcomes among similarly aged youth. Specifying a significance level of *α* = 0.05, power of 1 − *β* = 0.80, and an effect size of *d* = 0.2 multivariate analysis based on a similar study, requires a total sample of *n* = 199 to achieve 80% power.

In collaboration with a large Catholic school division in Alberta Canada, researchers recruited youth in grades 7–12 to participate in a three-month survey study to compare student mental health outcomes following the return to in-person learning and at 3-months follow-up. The school division allocated approximately 2000 eligible high school students for selection, of which 258 students (12.9%) volunteered to participate with parent/guardian consent and student assent.

### 2.2. Measures

We evaluated four mental health related outcomes: wellbeing, perceived stress (stress), attitudes toward help-seeking (help-seeking), and general mental health. We applied the World Health Organization Wellbeing Index (WHO-5) that contains five items measuring psychological wellbeing, with potential maximum scores of 30 and minimum scores of 5 (35). Higher scores indicate greater mental wellbeing. WHO-5 has been applied and validated internationally with good reliability and validity. WHO-5 had a coefficient of homogeneity of 0.52 to reflect the levels of wellbeing in adolescents (36). In the utilization for reflecting depression screening in adolescents, the WHO-5 had a diagnostic accuracy with the optimal cut-off score as 9, with the area under the receiver operating curve (AUC) of 0.87, a sensitivity of 0.74, and a specificity of 0.89 (37). We applied a validated perceived stress measure that contains a 10 item 5-point Likert scale; the minimum score of 0 indicates very little stress, and the maximum score of 40 indicates very high stress (38, 39). The Cronbach’s α reliability coefficient of the internal consistency was 0.89. The convergent validity was reflected by the high correlation with trait anxiety scales and the divergent validity was reflected by the non-significant correlations with scales of sensation seeking and aggression (38). To measure help-seeking attitudes, we evaluated student selections from five statements using a 7-point Likert scale. This measure was used in a previous study that indicated one factor of help-seeking intentions construct with the internal consistency reliability of *α* = 0.81 (39). Each response was assigned a value between 1 and 7; the range for total scores was between 5 and 35. Higher scores indicated greater positive attitudes and the scale demonstrated strong validity and reliability (39). We measured general mental health using the validated General Health Questionnaire (GHQ-12) (40) utilizing 10 statements measured on a 7-point Likert scale; we excluded 2 questions considered irrelevant to the school-context. The GHQ-12 had a one-dimensional factor with a reliability of Cronbach’s α coefficient = 0.90. Each participant received a total score of between 0 and 36, with higher scores indicating more severe health concerns. We further analyzed participants’ reported help-seeking sources over the past 3 months, such as parents, other family members, teachers, friends, health care providers and religious clerics. Participants were asked to rate their tendency and behaviors to seek help (1 = asked for help, 2 = wanted to but did not ask for help, and 3 = did not feel the need to ask for help).

### 2.3. Data analysis

We conducted descriptive statistical analysis with counts, percentages, means and standard deviations for continuous variables. Between baseline and follow-up, demographic differences such as gender (options were: male, female, other gender), grade level, English as a second language status (ESL), previous mental health service experiences, and access to school support-staff (helping staff) were compared using the Chi-square test. Participants were compared between baseline and follow-up using an independent t test. Two-way analysis of variance (ANOVA) was performed to analyze the effects of time-points (baseline and follow-up) and each demographic variable (grade, gender, ESL student, previous mental health service, and helping staff) on each outcome (wellbeing, perceived stress, attitudes toward help-seeking, and general mental health). Additionally, multiple linear regression analysis was conducted to predict students’ wellbeing based on the previously mentioned variables. Both were performed at baseline and follow-up, respectively. All analyses were conducted using SPSS software, with a significance level of *α* = 0.05. The significance level for comparing mental health outcomes between students of different genders was adjusted to *α* = 0.017 using the Bonferroni correction.

## 3. Results

A total of 258 school students at baseline and 132 at follow-up were enrolled in the study. The mean age and standard deviation of students at baseline was *M* = 14.05 and *SD* = 1.84, and at follow-up was *M* = 14.78 and *SD* = 1.79, respectively. Grade 7–9 students were considered “junior-high” students and grade 10–12 as “senior-high” or “high school” students. As illustrated in Table 1, Chi-square tests showed no significant enrollment differences between baseline and follow-up based on demographic characteristics (grade, gender, ESL students, previous mental health service, and helping staff).

**Table 1:** Demographic characteristics of school students.

		Baseline (*n* = 258)	Follow-up (*n* = 132)	*χ*^2^	*p*
Grade	Junior high	150	70	0.719	0.397
	Senior high	107	60		
Gender	Male	79	31	2.434	0.296
	Female	150	87		
	Unspecified gender	25	13		
ESL students	ESL	44	14	2.974	0.085
	Non-ESL	210	117		
Previous mental health service	Received mental health services	113	64	0.719	0.397
	Did not receive mental health services	142	67		
Helping staff	Had a school staff for help	165	91	1.071	0.301
	Did not have a school staff for help	92	40		

ESL students were students who self-identify as English as a Second Language learners. The item of helping staff represents whether there was a staff member at school that the student felt comfortable talking to when having a problem. *p-*values indicated baseline and follow-up differences.

### 3.1. Mental health status of school students at baseline and follow-up

Table 2 shows the average scores of students’ wellbeing, perceived stress, attitudes toward health seeking, and general mental health at baseline and follow-up. The independent sample t-test indicated that students at baseline and follow-up did not have significant differences across outcomes (*p*>0.05).Tables 3, 4 show the mean scores and standard deviations of each measure according to student-demographics at baseline and follow-up. Table 5 shows levels of student preference toward different help-seeking sources if/when previously faced with a mental health problem.

**Table 2:** Average scores of wellbeing, perceived stress, help seeking, and general mental health at baseline and follow-up.

	Baseline		Follow-up	
	Mean	SD	Mean	SD
Wellbeing	12.82	6.01	12.80	5.66
Perceived stress	21.73	4.12	22.29	2.65
Help-seeking	25.25	5.35	25.53	5.08
General mental health	14.46	6.87	14.76	6.71

**Table 3:** Average scores (standard deviation) of student mental health status at baseline by demographics.

		Grade (*n* = 257)			Gender (*n* = 254)				ESL students (*n* = 254)			Previous mental health (*n* = 255)			Helping staff (*n* = 257)		
	Total	Junior high	Senior high	*P*	Male	Female	Other	*P*	Yes	No	*P*	Yes	No	*P*	Yes	No	*P*
Wellbeing	12.82 (6.01)	13.96 (5.84)	11.25 (5.91)	<0.001	14.51 (5.75)	13.01 (5.93)	7.40 (3.49)	<0.001	12.59 (7.42)	12.87 (5.71)	0.82	11.28 (5.75)	14.10 (5.92)	<0.001	13.81 (5.81)	10.95 (5.96)	<0.001
Perceived stress	21.74 (4.12)	21.20 (4.21)	22.47 (3.89)	<0.05	20.27 (4.41)	22.34 (3.80)	22.57 (4.05)	<0.001	21.44 (3.50)	21.83 (4.25)	0.60	22.72 (3.91)	20.89 (4.09)	<0.001	22.02 (4.04)	21.22 (4.23)	0.15
Help-seeking	25.25 (5.35)	25.12 (5.54)	25.43 (5.10)	0.67	25.80 (5.47)	25.38 (5.40)	23.35 (4.49)	0.16	26.41 (4.10)	25.07 (5.55)	0.09	25.58 (5.60)	24.99 (5.20)	0.41	26.79 (5.07)	22.32 (4.62)	<0.001
General mental health	14.46 (6.87)	12.90 (6.50)	16.61 (6.82)	<0.001	11.79 (6.14)	14.63 (6.67)	19.87 (5.89)	<0.001	13.34 (7.70)	14.62 (6.67)	0.30	16.23 (6.74)	12.98 (6.58)	<0.001	13.64 (6.85)	16.15 (6.63)	<0.01

In this table and Table 4, *P-*values demonstrated student mental health outcomes’ differences between the subgroups of each demographic category.

**Table 4:** Average scores (standard deviation) of student mental health status at follow-up by demographics.

		Grade (*n* = 130)			Gender (*n* = 131)				ESL students (*n* = 131)			Previous mental health (*n* = 131)			Helping staff (*n* = 131)		
	Total	Junior high	Senior high	*P*	Male	Female	Other	*P*	Yes	No	*P*	Yes	No	*P*	Yes	No	*P*
Wellbeing	12.80 (5.66)	14.36 (5.84)	10.97 (4.97)	<0.001	14.19 (5.43)	13.01 (5.64)	7.77 (3.90)	<0.01	11.00 (6.48)	13.04 (5.56)	0.20	10.77 (5.45)	14.61 (5.19)	<0.001	13.81 (5.57)	10.48 (5.32)	<0.01
Perceived stress	22.29 (2.65)	22.38 (2.40)	22.34 (2.84)	0.92	21.62 (2.53)	22.61 (2.73)	21.85 (2.34)	0.19	22.00 (2.66)	22.31 (2.66)	0.69	22.31 (3.04)	22.28 (2.24)	0.95	22.48 (2.66)	21.86 (2.63)	0.24
Help-seeking	25.53 (5.08)	25.67 (4.85)	25.20 (5.24)	0.63	25.00 (5.13)	25.46 (4.97)	27.08 (6.02)	0.50	25.62 (4.21)	25.58 (5.18)	0.98	26.04 (5.32)	25.03 (4.83)	0.29	25.85 (5.18)	24.76 (4.81)	0.30
General mental health	14.76 (6.71)	14.05 (6.85)	15.56 (6.60)	0.24	16.29 (7.47)	14.53 (6.39)	13.00 (7.40)	0.35	16.54 (5.13)	14.54 (6.91)	0.32	14.96 (6.60)	14.56 (6.89)	0.75	14.01 (6.72)	16.61 (6.44)	0.06

**Table 5:** Most practiced help-seeking sources.

Help-seeking sources (baseline, *n* = 258)	*n*	%	Help-seeking sources (follow-up, *n* = 132)	*n*	%
Parents/Guardians	98	42.1	Parents/Guardians	54	45.4
Close friends	91	40.3	Close friends	51	43.6
Mental health professionals	50	22.9	Mental health professionals	27	24.3
Teachers/counselors/coaches	47	18.2	Teachers/counselors/coaches	26	22.6
Siblings	36	16.9	School health professionals	18	15.8
School health professionals	32	14.7	Family physicians	15	13.4
Family physicians	26	12.3	Siblings	14	12.4

The figures indicated the number and percentages of students who opted to “ask for help” from those persons who were regarded as help-seeking sources.

### 3.2. Demographic differences for student mental health status at baseline and follow-up

Table 6 presents demographic differences on each outcome. When comparing grade-level differences, it showed that there were not significant differences in wellbeing (*p* = 0.93), general mental health (*p* = 0.95), stress (*p* = 0.20), and help-seeking (*p* = 0.79) from baseline to 3 months follow-up. The senior high students had lower wellbeing [*F*(1, 371) = 23.71, *p* < 0.001] and worse general mental health [*F*(1, 338) = 11.48, *p* < 0.001] than junior high students. Their stress (*p* = 0.14) and help-seeking (*p* = 0.90) were not significantly different. There were no significant interaction effects between time and grade on each outcome wellbeing (*p* = 0.59), stress (*p* = 0.11), help-seeking (*p* = 0.53), and general mental health (*p* = 0.15), indicating that the change in each outcome over time and the differences in each outcome among different grade levels was not significantly influenced by each other.

**Table 6:** Two-way ANOVA results (demographic differences for student mental health at baseline and follow-up).

	Wellbeing			Perceived stress			Help-seeking			General mental health		
	*F*	*p*	η2	*F*	*p*	η2	*F*	*p*	η2	*F*	*p*	η2
Grade (*n* = 257)												
Main effect for time point	0.008	0.928	<0.001	1.653	0.199	0.005	0.069	0.792	<0.001	0.003	0.954	<0.001
Main effect for grade	23.709	**<0.001**	0.060	2.231	0.136	0.006	0.017	0.896	<0.001	11.475	**<0.001**	0.033
Interaction effect	0.300	0.585	0.001	2.573	0.110	0.007	0.405	0.525	0.001	2.055	0.153	0.006
Gender (*n* = 254)												
Main effect for time point	0.001	0.982	<0.001	0.332	0.565	0.001	1.621	0.204	0.005	0.699	0.404	0.002
Main effect for gender	18.353	**<0.001**	0.091	5.320	**0.005**	0.030	0.021	0.980	<0.001	1.490	0.227	0.009
Interaction effect	0.052	0.950	<0.001	1.142	0.320	0.006	2.103	0.124	0.012	8.470	**<0.001**	0.048
ESL students (*n* = 254)												
Main effect for time point	0.524	0.469	0.001	0.716	0.398	0.002	0.025	0.875	<0.001	1.761	0.185	0.005
Main effect for ESL students	1.423	0.234	0.004	0.317	0.574	0.001	0.577	0.448	0.002	0.094	0.760	<0.001
Interaction effect	0.811	0.369	0.002	0.005	0.946	<0.001	0.526	0.469	0.002	1.952	0.163	0.006
Previous mental health service (*n* = 255)												
Main effect for time point	<0.001	0.999	<0.001	1.488	0.223	0.004	0.168	0.682	<0.001	0.042	0.838	<0.001
Main effect for previous mental health service	29.225	**<0.001**	0.073	5.326	**0.022**	0.015	1.741	0.188	0.005	5.614	**0.018**	0.016
Interaction effect	0.700	0.403	0.002	4.958	**0.027**	0.014	0.116	0.734	<0.001	3.404	0.066	0.010
Helping staff (*n* = 257)												
Main effect for time point	0.128	0.721	<0.001	1.579	0.210	0.004	1.511	0.220	0.004	0.246	0.620	0.001
Main effect for helping staff	21.596	**<0.001**	0.055	2.587	0.109	0.007	20.649	**<0.001**	0.056	9.197	**0.003**	0.026
Interaction effect	0.129	0.720	<0.001	0.047	0.828	<0.001	7.654	**0.006**	0.022	0.002	0.961	<0.001

The main effect for time point refers to whether there was a significant difference of the outcome variable between baseline and follow-up. The main effect for a demographic variable refers to whether there was a significant difference of the outcome variable within the demographic group, for example, between junior and senior high students. The interaction effect refers to whether there was a significant interaction between time-point and the demographic variable on an outcome variable in a specific demographic group. The bold values suggested a significant main effect or interaction effect.

The gender analysis showed that there were not significant changes in wellbeing (*p* = 0.98), general mental health (*p* = 0.40), stress (*p* = 0.57), and help-seeking (*p* = 0.20) from baseline to follow-up. The gender unspecified students had lower wellbeing than male students and female students (*p* < 0.001). The male students had less stress than female students (*p* >0.001). There were no significant differences in stress found between gender unspecified students and male (*p* = 0.05) or female students (*p* = 0.91). The three groups of students did not differ in their attitudes toward help-seeking (*p* = 0.98) and general mental health (*p* = 0.23). The interactions were not significant between time and gender on wellbeing (*p* = 0.95), stress (*p* = 0.32) and attitudes toward help-seeking (*p* = 0.12). The interaction was significant between time and gender on general mental health (*p* < 0.001). Further analysis showed that the male students’ general mental health worsened at follow-up compared to baseline [*F*(1, 333) = 8.36, *p* < 0.01]. The students who did not specify a gender showed better general mental health at follow-up than baseline [*F*(1, 333) = 8.70, *p* < 0.01]. The female students’ general mental health did not differ from baseline to follow-up [*F*(1, 333) = 0.01, *p* = 0.92]. At baseline, male students had better general mental health than female students [*F*(1, 333) = 13.44, *p* < 0.05] and students who did not specify a gender (*p* < 0.001), while female students had better general mental health than students who did not specify a gender (*p* < 0.001). General mental health at follow-up was not significantly different between gender demographics, comparing male against female students [*F*(1, 333) = 1.14, *p* = 0.75], and male or female against students of unspecified gender (*p* = 0.47, *p* = 1.00).

Analysis of ESL students versus non-ESL students showed no significant differences in wellbeing (*p* = 0.47), stress (*p* = 0.40), help-seeking (*p* = 0.88), general mental health (*p* = 0.19) between baseline and follow-up. The ESL and non-ESL students did not differ significantly in their wellbeing (*p* = 0.23), stress (*p* = 0.57), help-seeking (*p* = 0.45), and general mental health (*p* = 0.76). There were no significant interaction effects between time and ESL student on each outcome variable (*p* = 0.37, *p* = 0.95, *p* = 0.47, *p* = 0.16). This indicates that the change in each outcome over time and the differences in each outcome among ESL or non-ESL student was not significantly influenced by each other.

Analysis of previous mental health service experience versus no prior experience showed no significant changes in wellbeing (*p* = 0.99), general mental health (*p* = 0.84), stress (*p* = 0.22), attitudes toward help-seeking (*p* = 0.68) between baseline and follow-up. Students who received previous mental health service had lower wellbeing [*F*(1, 370) = 29.23, *p* < 0.001], lower general mental health [*F*(1, 337) = 5.61, *p* < 0.05], and higher stress [*F*(1, 352) = 5.33, *p* < 0.05] than students without prior experience accessing mental health services. They did not differ in help-seeking [*F*(1, 344) = 1.74, *p* = 0.19]. The interaction between time and previous mental health service experience was not significant for wellbeing (*p* = 0.40), attitudes toward help-seeking (*p* = 0.73), and general mental health (*p* = 0.07), but was significant for stress (*p* < 0.05). Further analysis showed that at baseline, students who received previous mental health services had comparatively higher stress than those who did not [*F*(1, 352) = 14.86, *p* < 0.001]. However, this did not generalize to the follow-up time point [*F*(1, 352) = 0.003, *p* = 0.96]. In addition, students who did not receive previous mental health services reported greater stress at follow-up than baseline [*F*(1, 352) = 6.20, *p* < 0.05].

Analysis of having access to school-support staff (helping staff) versus no access did not identify significant differences in wellbeing (*p* = 0.72), general mental health (*p* = 0.62), stress (*p* = 0.21), and attitudes toward help-seeking (*p* = 0.22) between baseline and follow-up. The students who had access to support-staff had higher positive wellbeing [*F*(1, 372) = 21.60, *p* < 0.001], higher general mental health [*F*(1, 339) = 9.20, *p* < 0.05] and help-seeking attitudes [*F*(1, 346) = 20.65, *p* < 0.001] than those who did not. They did not differ in stress [*F*(1, 355) = 2.59, *p* = 0.11]. The interaction between time and access to school-support staff was not significant on wellbeing (*p* = 0.72), stress (*p* = 0.83), general mental health (*p* = 0.96), but was significant for help-seeking attitudes (*p* < 0.01). That is, students who did not have access to support staff showed improved help-seeking attitudes at follow-up from baseline [*F*(1, 346) = 5.80, *p* < 0.05]. Those students who had access to support staff did not differ in help-seeking attitudes over time (*F*(1, 346) = 1.90, *p* = 0.17). At baseline, the students who had support-staff showed greater help-seeking attitudes than students without support-staff [*F*(1, 346) = 42.98, *p* < 0.001]. They did not differ in help-seeking attitudes at follow-up [*F*(1, 346) = 1.15, *p* = 0.29].

### 3.3. Regression analyses for wellbeing and general mental health at baseline and follow-up

#### 3.3.1. Baseline

Students’ better wellbeing at baseline was predicted by their more positive help-seeking attitudes, less perceived stress, grade, and previous mental health services utilization, which explained 38.1% of the variance in student’s wellbeing, *F*(4, 198) = 30.51, *p* < 0.001, with an *R*^2^ of 0.381. Students’ predicted wellbeing was equal to 7.83 + 0.56 (help-seeking attitudes) – 2.69 (grade) – 2.08 (previous mental health services) – 0.17 (perceived stress). For every one-unit increase in positive help-seeking attitudes, the predicted wellbeing was expected to increase by 0.56 units and for every one-unit increase in perceived stress, the predicted wellbeing was expected to decrease by 0.17 units. Senior high students had 2.69 units of wellbeing less than junior high student and students who received previous mental health services had 2.08 units of wellbeing less than students who did not.

Students’ better general mental health at baseline was predicted by their higher wellbeing, less perceived stress, and gender, which explained 60.3% of the variance in their general mental health, *F*(3, 189) = 95.78, *p* < 0.001, with an *R*^2^ of 0.603. Students’ predicted general mental health was equal to 19.45–0.80 (wellbeing) + 1.69 (gender) + 0.18 (perceived stress). For every one-unit decrease in wellbeing, the predicted general mental health scores increased (students’ general mental health worsened) by 0.80 units and for every one-unit increase in perceived stress, the predicted general mental health scores increased by 0.18 units. Students of different genders had 1.69 units differences in general mental health.

#### 3.3.2. Follow-up

At follow-up, students’ better wellbeing was predicted by their more perceived stress, previous mental health services utilization, grade, and having access to support staff, which accounted for 24.6% of variance in their wellbeing, *F*(4, 95) = 7.768, *p* < 0.001, with an *R*^2^ of 0.246. Students’ predicted wellbeing was equal to 7.58–3.39 (previous mental health services) – 2.74 (grade) + 2.78 (having access to support staff) + 0.40 (perceived stress). For each one-unit increase in stress, the predicted wellbeing increased by 0.40 units. Students who received previous mental health services had 3.39 units of wellbeing less than those who did not. Senior high students had 2.74 units of wellbeing less than junior high students and students who had access to support staff had 2.78 units of wellbeing more than those who did not.

Students’ more perceived stress predicted their better general mental health and accounted for 9% of variance in general mental health [*F*(1, 97) = 9.42, *p* < 0.01, with an *R*^2^ of 0.09]. Students’ predicted general mental health was equal to 21.87–0.75 (perceived stress). For each one-unit decrease in stress, the predicted general meatal scores increased by 0.75 units.

## 4. Discussion

Our study indicated that children and adolescents exhibited relatively stable mental health and wellbeing status during their reintegration with in-person classroom learning over time following pandemic-related cycles of school-closures. Specific demographic groups of students experienced declines in their mental health and wellbeing status after returning to school for 3 months, which requires further interpretation and attention from schools, mental health care system and researchers. Furthermore, our findings highlighted the benefits of students’ surrounding social support resources and their positive attitudes toward seeking help. These factors served as buffers against perceived stress and contributed to an improvement in their mental health and wellbeing. Over the 3-month period of in-person learning at school, positive adaptation and coping emerged among the participating students, as evidenced by a positive relationship between stress and general mental health or wellbeing. This implies that their stress responses to public health event was enhanced with positive stress coping and social supports.

Upon returning to in-person learning and at 3-months follow-up, senior-high students had lower wellbeing and lower general mental health than junior high students. Students who did not specify a gender had the lowest scores on wellbeing at both time points compared to male and female students. In general, these results are consistent with previous studies which were conducted among children and adolescents of similar age range (26) and align with recommendations by international organizations emphasizing the importance of interventions to meet the needs of diverse youth groups (41) while mitigating mental health degradation following grand-scale adverse life-experiences (42). Some researchers found that in pre-adolescent children, their younger subgroup was more vulnerable to experiencing adverse mental health outcomes due to the pandemic than their older counterparts (43). These discrepancies may be due to differences in geographic location, social and cultural perspectives, or tools used to measure mental health. More research is required to understand the apparent disparity between conflicting results in the body of literature.

We further analyzed participants’ mental health based on their access to, and preferences for supports such as school support-staff and help-providing parents. Students who had access to school support-staff reported higher wellbeing, better help-seeking attitudes, and better general mental health than those who did not. This result is consistent with previous studies espousing social support within schools may have improved child and adolescent mental health outcomes during the pandemic (2, 10).

When comparing longitudinally from baseline to follow-up within groups, male students experienced decreased general mental health and students who did not receive previous mental health services reported more stress, yet the students who did not specify a gender showed improved general mental health and students who did not have access to support-staff reported improved help-seeking attitudes. This may suggest that although male students and students without previous mental health service demonstrated overall more positive mental health than other students, the fact that their mental health declined overtime may warrant additional attention to support their long-term mental health. The most-preferred help-seeking sources were similar at both survey time points; with parents, guardians, and close friends as the most-preferred help sources, followed by mental health professionals, teachers, counselors, and coaches. We also investigated the factors predicting students’ wellbeing and general mental health over time, ultimately finding a mix of shared and distinct factors. Given these results, it is clear that a students’ family and broader community play important roles in their mental health.

In the present study, parents and guardians were valued as the most preferred sources for help at baseline and at 3-months follow-up. Although we did not follow-up on the quality of help students received from their parents, it highlights the importance of positive communications between youth and parents that were protective factors for youth mental health in home quarantine (9). Other preferred sources of help including teachers, friends, and peers, are speculated to have been protective factors for children and adolescents’ mental health during the pandemic (2). Interestingly, we found that students who received previous mental health services reported lower wellbeing, more perceived stress, and worse general mental health at baseline and lower wellbeing at follow-up than those who did not. This result suggests that students who had received previous mental health services experienced persistent risk for mental health problems and greater difficulties transitioning to in-person learning. This result is also consistent with a large-scale Canadian survey during the pandemic that showed children and youth with existing mental illness experienced deteriorating mental health and it further pointed out that enhanced social interactions may be a strong protective factor to improve mental health outcomes (44).

In the past, the validated WHO-5, was also used for depression screening. According to a previous survey using WHO-5 as a clinical screening tool (37), a cut-off score of 9 demonstrated good diagnostic accuracy for adolescent depression. In the current study, the general average scores of students’ wellbeing at baseline and follow-up were around 12, above the cut-off score of 9 for depression screening. This may further support our finding that this cohort of students demonstrated relatively stable mental health between baseline and follow-up. Although some previous international studies suggest that children and adolescents reported more mental health problems upon returning to school during the pandemic (10, 31), our finding of relatively stable mental health among students is consistent with earlier Canadian-conducted surveys to indicate that student-stress incurred by the COVID-19 pandemic did not lead to significant concerns about mental health problems such as chronic stress and negative affect (25, 26). However, for specific subgroups, such as students who did not indicate a gender and students who did not have support staff, their wellbeing mean scores were generally below 12 and close to the cut-off of 9, suggesting possible benefit of additional help to these students.

At baseline, students who reported greater positive help-seeking attitudes, less experience accessing mental health services, less perceived stress, and lower grade-levels were more-likely to experience greater wellbeing during their initial return to school. After 3 months, wellbeing was predicted by less experience accessing mental health services, more perceived stress, having support-staff, and grade levels. Greater general mental health at baseline was predicted by greater wellbeing, less perceived stress, and gender; and at follow-up was only predicted by greater perceived stress. Notably, students’ greater wellbeing and general mental health were predicted by less perceived stress at baseline but by more perceived stress at follow-up. This may indicate that students’ perceived stress affects their wellbeing and general mental health shifted along with their transition to in-person school learning. This suggests that stress became less risky for mental health problems but rather a “eustress” (5) for students who returned to in-person learning. Previous research has found eustress and positive stress to be associated with increased wellbeing in adolescents (45). Moreover, at 3-months follow-up, students’ wellbeing was no longer predicted by their help-seeking attitudes but instead by having access to support staff. This may suggest that help-seeking attitudes played an essential role in affecting students’ wellbeing during distance-learning experiences. Contrastingly, external environmental factors such as support-staff became highly predictive of wellbeing after students returned to in-person learning.

Therefore, providing students, parents, friends and stakeholders around children and youth, such as teachers, with mental health education may be a strategy to support youth mental health. Research has identified how improving educators’ mental health literacy can enhance the early identification of mental health problems and referral of students in need of help to appropriate mental health care (46, 47). Also embedding mental health literacy in the classroom teaching has demonstrated its effectiveness in improving students’ capacity to take care of their own mental health and the mental health of people they care about (34, 48, 49). Parent mental health literacy interventions are emerging, however, there are limited studies with mixed quality to determine their overall effectiveness (50). Students’ eustress about their wellbeing and general mental health implies that positive perspectives about stress may benefit students in the context of school-based mental health (45). A recent study showed that contextualizing stress positively may lead to the reduction of perceived stress among youth (39). This finding may also benefit the professional development of teachers in fostering eustress and positive mental health in the classroom. Generally, our findings may help related stakeholders understand student mental health and wellbeing to proactively inform education, health promotion, and intervention supports. For example, educators, researchers and decision makers in Canada have started the conversation and work on how to align mental health education in the classroom with educational policies at local, provincial and national levels to support student mental health (51, 52).

### 4.1. Limitations

There are a few limitations of this study. Participants were students in a Catholic school division in Alberta, Canada. As such, the results may not be generalized to other school-affiliations or geographic locations. Additionally, we applied self-report measures among students without parents, guardians, or teachers’ contributing their insights to the survey data, which may lead to biased interpretations. Though our sample size was sufficient to complete the statistical analysis, a larger cohort of students would have provided more data and more favorable significance and power. Because 12.9% of invited students agreed to participate in the study, there is a risk of self-selection bias impacting outcomes. Lastly, we did not collect qualitative data about student-experience to corroborate our interpretations of the quantitative data.

## 5. Conclusion and implications

The present study illustrated school students’ mental health representation, psychological distress, social support, and sources of preferred help upon re-entering schools for in-person learning and at 3-months follow-up. The mental health of this cohort of students was relatively stable during the transition back to in-person learning. However, the mental health of specific subgroups such as females, students who did not specify a gender, adolescents of higher grade levels, and students who previously utilized mental health services was not as positive as their counterparts. This suggests a potential need for further support and targeted research. In addition, male students, students who did not specify a gender, students who did not receive previous mental health services and those who did not have support-staff were more likely to experience fluctuations of their mental health while transitioning back to in-person learning. Positive attitudes about preferred sources of help, such as parents and friends, in addition to the presence of support staff, were predictive of greater student mental health upon their return to in-person learning. Outcomes may be useful in determining how-best to equip teachers and health care professionals to support students following public health emergencies that affect access to in-person experiences.

## Data availability statement

The raw data supporting the conclusions of this article will be made available by the authors, without undue reservation.

## Ethics statement

The studies involving humans were approved by the University of Alberta Ethics Research Board 2. The studies were conducted in accordance with the local legislation and institutional requirements. Written informed consent for participation in this study was provided by the participants’ legal guardians/next of kin.

## Author contributions

YW, CS, AB, AG, and BC conceptualized the study design, supervised the study operation, data collection and analysis, and contributed significantly to the manuscript preparation. LQ and RM cleaned the data, conducted data analysis, and prepared the manuscription under supervision. PS, HP, AB, and RM contributed significantly to provide feedback on study design, prepare and finalize the manuscript. All authors contributed to the article and approved the submitted version.

## Funding

This study was supported as part of the Women and Children’s Health Research Institute Innovation Grant at the University of Alberta.

## Conflict of interest

The authors declare that the research was conducted in the absence of any commercial or financial relationships that could be construed as a potential conflict of interest.

## Publisher’s note

All claims expressed in this article are solely those of the authors and do not necessarily represent those of their affiliated organizations, or those of the publisher, the editors and the reviewers. Any product that may be evaluated in this article, or claim that may be made by its manufacturer, is not guaranteed or endorsed by the publisher.

## References

[1] United Nations Educational, Scientific and Cultural Organization. COVID-19 impact on education. (2022). Available at: https://webarchive.unesco.org/web/20220629024039/, https://en.unesco.org/covid19/educationresponse/ (Accessed December 19, 2022).

[2] Jones EA Mitra AK Bhuiyan AR. Impact of COVID-19 on mental health in adolescents: a systematic review. Int J Environ Res Public Health. (2021) 18:2470. doi: 10.3390/ijerph18052470, PMID: 33802278 PMC7967607

[3] Dumas TM Ellis W Litt DM. What does adolescent substance use look like during the COVID-19 pandemic? Examining changes in frequency, social contexts, and pandemic-related predictors. J Adolesc Health. (2020) 67:354–61. doi: 10.1016/j.jadohealth.2020.06.018, PMID: 32693983 PMC7368647

[4] Zhang C Ye M Fu Y Yang M Luo F Yuan J. The psychological impact of the COVID-19 pandemic on teenagers in China. J Adolesc Health. (2020) 67:747–55. doi: 10.1016/j.jadohealth.2020.08.026, PMID: 33041204 PMC7543885

[5] Selye HA. History and general outline of the stress concept In H Selye editor: Stress in health and disease. Boston, MA: Butterworths, Inc (1976). 3–4.

[6] Cohen S Wills TA. Stress, social support, and the buffering hypothesis. Psychol Bull. (1985) 98:310–57. doi: 10.1037/0033-2909.98.2.310 3901065

[7] Compas BE. Psychobiological processes of stress and coping: implications for resilience in children and adolescents—comments on the papers of Romeo & McEwen and fisher et al. Ann N Y Acad Sci. (2006) 1094:226–34. doi: 10.1196/annals.1376.024, PMID: 17347354

[8] Schwarzer R Knoll N. Functional roles of social support within the stress and coping process: a theoretical and empirical overview. Int J Psychol. (2007) 42:243–52. doi: 10.1080/00207590701396641

[9] Panchal U Salazar de Pablo G Franco M Moreno C Parellada M Arango C. The impact of COVID-19 lockdown on child and adolescent mental health: systematic review. Eur Child Adolesc Psychiatry. (2021) 32:1151–77. doi: 10.1007/s00787-021-01856-w, PMID: 34406494 PMC8371430

[10] Widnall E Winstone L Plackett R Adams EA Haworth CM Mars B. Impact of school and peer connectedness on adolescent mental health and well-being outcomes during the COVID-19 pandemic: a longitudinal panel survey. Int J Environ Res Public Health. (2022) 19:6768. doi: 10.3390/ijerph19116768 35682350 PMC9180617

[11] Vidal C Holland ET Duriseti RS. Rejoinder 3: school closures: the trigger point in the decline in pediatric mental health outcomes during the COVID-19 pandemic. J Can Acad Child Adolesc Psychiatry. (2023) 32:88–92. PMID: 37181439 PMC10168615

[12] Detsky AS Bogoch II. COVID-19 in Canada: experience and response. JAMA. (2020) 324:743–4. doi: 10.1001/jama.2020.14033 32790824

[13] Razak F Shin S Naylor CD Slutsky AS. Canada’s response to the initial 2 years of the COVID-19 pandemic: a comparison with peer countries. CMAJ. (2022) 194:E870–7. doi: 10.1503/cmaj.220316, PMID: 35760433 PMC9332918

[14] Azevedo JP Rogers FH Ahlgren SE Cloutier M Chakroun B Chang G. The state of the global education crisis: a path to recovery (English). Washington, DC: World Bank Group. (2021). Available at: http://documents.worldbank.org/curated/en/416991638768297704/The-State-of-the-Global-Education-Crisis-A-Path-to-Recovery (Accessed August 3, 2023).

[15] Krishnaratne S Littlecott H Sell K Burns J Rabe JE Stratil JM. Measures implemented in the school setting to contain the COVID-19 pandemic. Cochrane Database Syst Rev. (2022) 2022:CD015029. doi: 10.1002/14651858.CD015029, PMID: 35037252 PMC8762709

[16] Wang S Chen L Ran H Che Y Fang D Sun H. Depression and anxiety among children and adolescents pre and post COVID-19: a comparative meta-analysis. Front Psych. (2022) 13:1752. doi: 10.3389/fpsyt.2022.917552, PMID: 35990058 PMC9381924

[17] Liang L Ren H Cao R Hu Y Qin Z Li C. The effect of COVID-19 on youth mental health. Psychiatry Q. (2020) 91:841–52. doi: 10.1007/s11126-020-09744-3, PMID: 32319041 PMC7173777

[18] Cluver L Lachman JM Sherr L Wessels I Krug E Rakotomalala S. Parenting in a time of COVID-19. Lancet. (2020) 395:e64. doi: 10.1016/S0140-6736(20)30736-4, PMID: 32220657 PMC7146667

[19] Jiao WY Wang LN Liu J Fang SF Jiao FY Pettoello-Mantovani M. Behavioral and emotional disorders in children during the COVID-19 epidemic. J Pediatr. (2020) 221:264–266.e1. doi: 10.1016/j.jpeds.2020.03.013, PMID: 32248989 PMC7127630

[20] Lee J. Mental health effects of school closures during COVID-19. Lancet Child Adolesc Health. (2020) 4:421. doi: 10.1016/S2352-4642(20)30109-7, PMID: 32302537 PMC7156240

[21] Statistics Canada. Canadians report lower self-perceived mental health during the_COVID-19 pandemic. (2020). Available at: https://www150.statcan.gc.ca/n1/pub/45-28-0001/2020001/article/00003-eng.htm (Accessed December 16, 2022).

[22] Seddighi H Salmani I Javadi MH Seddighi S. Child abuse in natural disasters and conflicts: a systematic review. Trauma Violence Abuse. (2021) 22:176–85. doi: 10.1177/1524838019835973, PMID: 30866745

[23] Carroll N Sadowski A Laila A Hruska V Nixon M Ma DW. The impact of COVID-19 on health behavior, stress, financial and food security among middle to high income Canadian families with young children. Nutrients. (2020) 12:2352. doi: 10.3390/nu12082352, PMID: 32784530 PMC7468859

[24] Angus Reid Institute. Worry, gratitude and boredom: as COVID-19 affects mental, financial health, who fares worse? (2020). Available at: http://angusreid.org/covid19-mental-health/ (Accessed December 19, 2022).

[25] Angus Reid Institute. Kids AND COVID-19: Canadian children are done with school from home, fear falling behind, and miss their friends. (2020). Available at: https://angusreid.org/COVID19-kids-opening-schools/ (Accessed December 19, 2022).

[26] Schwartz KD Exner-Cortens D McMorris CA Makarenko E Arnold P Van Bavel M. COVID-19 and student well-being: stress and mental health during return-to-school. Can J Sch Psychol. (2021) 36:166–85. doi: 10.1177/08295735211001653, PMID: 34040284 PMC8114331

[27] Meherali S Punjani N Louie-Poon S Abdul Rahim K Das JK Salam RA. Mental health of children and adolescents amidst COVID-19 and past pandemics: a rapid systematic review. Int J Environ Res Public Health. (2021) 18:3432. doi: 10.3390/ijerph18073432, PMID: 33810225 PMC8038056

[28] Racine N McArthur BA Cooke JE Eirich R Zhu J Madigan S. Global prevalence of depressive and anxiety symptoms in children and adolescents during COVID-19: a meta-analysis. JAMA Pediatr. (2021) 175:1142–50. doi: 10.1001/jamapediatrics.2021.2482, PMID: 34369987 PMC8353576

[29] Silverstone PH Bercov M Suen VY Allen A Cribben I Goodrick J. Long-term results from the empowering a multimodal pathway toward healthy youth program, a multimodal school-based approach, show marked reductions in suicidality, depression, and anxiety in 6,227 students in grades 6–12 (aged 11–18). Front Psych. (2017) 8:81. doi: 10.3389/fpsyt.2017.00081, PMID: 28555115 PMC5430037

[30] Silverstone PH Bercov M Suen VY Allen A Cribben I Goodrick J. Initial findings from a novel school-based program, EMPATHY, which may help reduce depression and suicidality in youth. PLoS One. (2015) 10:e0125527. doi: 10.1371/journal.pone.0125527, PMID: 25974146 PMC4431804

[31] Zhang L Zhang D Fang J Wan Y Tao F Sun Y. Assessment of mental health of Chinese primary school students before and after school closing and opening during the COVID-19 pandemic. JAMA Netw Open. (2020) 3:e2021482. doi: 10.1001/jamanetworkopen.2020.21482, PMID: 32915233 PMC7489803

[32] Wang L Zhang Y Chen L Wang J Jia F Li F. Psychosocial and behavioral problems of children and adolescents in the early stage of reopening schools after the COVID-19 pandemic: a national cross-sectional study in China. Transl Psychiatry. (2021) 11:342. doi: 10.3390/ijerph19116768, PMID: 34083509 PMC8172553

[33] Qu Y Li X Ni B He X Zhang K Wu G. Identifying the role of parent–child conflict and intimacy in Chinese adolescents’ psychological distress during school reopening in COVID-19 pandemic. Dev Psychol. (2021) 57:1735–47. doi: 10.1037/dev0001218, PMID: 34807693

[34] Milin R Kutcher S Lewis SP Walker S Wei Y Ferrill N. Impact of a mental health curriculum on knowledge and stigma among high school students: a randomized controlled trial. J Am Acad Child Adolesc Psychiatry. (2016) 55:383–391.e1. doi: 10.1016/j.jaac.2016.02.018, PMID: 27126852

[35] Topp CW Østergaard SD Søndergaard S Bech P. The WHO-5 well-being index: a systematic review of the literature. Psychother Psychosom. (2015) 84:167–76. doi: 10.1159/000376585, PMID: 25831962

[36] Blom EH Bech P Högberg G Larsson JO Serlachius E. Screening for depressed mood in an adolescent psychiatric context by brief self-assessment scales–testing psychometric validity of WHO-5 and BDI-6 indices by latent trait analyses. Health Qual Life Outcomes. (2012) 10:1–6. doi: 10.1186/1477-7525-10-149, PMID: 23227908 PMC3575311

[37] Allgaier AK Pietsch K Frühe B Prast E Sigl-Glöckner J Schulte-Körne G. Depression in pediatric care: is the WHO-five well-being index a valid screening instrument for children and adolescents? Gen Hosp Psychiatry. (2012) 34:234–41. doi: 10.1016/j.genhosppsych.2012.01.007, PMID: 22325631

[38] Roberti JW Harrington LN Storch EA. Further psychometric support for the 10-item version of the perceived stress scale. J Coll Couns. (2006) 9:135–47. doi: 10.1002/j.2161-1882.2006.tb00100.x

[39] Wei Y Kutcher S Austen E Comfort A Gilham C MacDougall C. The impact of transitions, a mental health literacy intervention with embedded life skills for postsecondary students: preliminary findings from a naturalistic cohort study. Can J Psychiatry. (2022) 67:452–61. doi: 10.1177/07067437211037131, PMID: 34379024 PMC9152239

[40] Hankins M. The reliability of the twelve-item general health questionnaire (GHQ-12) under realistic assumptions. BMC Public Health. (2008) 8:1–7. doi: 10.1186/1471-2458-8-355, PMID: 18854015 PMC2572064

[41] Imran N Zeshan M Pervaiz Z. Mental health considerations for children and adolescents in COVID-19 pandemic. Pak J Med Sci. (2020) 36:S67–72. doi: 10.12669/pjms.36.COVID19-S4.2759, PMID: 32582317 PMC7306970

[42] Brown MR Pazderka H Agyapong VI Greenshaw AJ Cribben I Brett-MacLean P. Mental health symptoms unexpectedly increased in students aged 11–19 years during the 3.5 years after the 2016 Fort McMurray wildfire: findings from 9,376 survey responses. Front Psych. 12:676256. doi: 10.3389/fpsyt.2021.676256, PMID: 34093284 PMC8172807

[43] Ravens-Sieberer U Kaman A Erhart M Devine J Schlack R Otto C. Impact of the COVID-19 pandemic on quality of life and mental health in children and adolescents in Germany. Eur Child Adolesc Psychiatry. (2022) 31:879–89. doi: 10.1007/s00787-021-01726-5, PMID: 33492480 PMC7829493

[44] Cost KT Crosbie J Anagnostou E Birken CS Charach A Monga S. Mostly worse, occasionally better: impact of COVID-19 pandemic on the mental health of Canadian children and adolescents. Eur Child Adolesc Psychiatry. (2021) 31:671–4. doi: 10.1007/s00787-021-01744-3, PMID: 33638005 PMC7909377

[45] Branson V Palmer E Dry MJ Turnbull D. A holistic understanding of the effect of stress on adolescent well-being: a conditional process analysis. Stress Health. (2019) 35:626–41. doi: 10.1002/smi.2896, PMID: 31469222

[46] Baxter A Wei Y Kutcher S Cawthorpe D. School-based mental health literacy training shifts the quantity and quality of referrals to tertiary child and adolescent mental health services: a Western Canada regional study. PLoS One. (2022) 17:e0277695. doi: 10.1371/journal.pone.0277695, PMID: 36378651 PMC9665371

[47] Wei Y Kutcher S Baxter A Heffernan A. The program evaluation of 'Go-to educator Training' on educators' knowledge about and stigma toward mental illness in six Canadian provinces. Early Interv Psychiatry. (2021) 15:922–31. doi: 10.1111/eip.13037, PMID: 32893458

[48] Simkiss NJ Gray NS Kemp AH Dunne C Snowden RJ. A randomised controlled trial evaluating the guide Cymru mental health literacy intervention programme in year 9 (age 13–14) school pupils in Wales. BMC Public Health. (2023) 23:1062. doi: 10.1186/s12889-023-15922-2, PMID: 37277757 PMC10239719

[49] Wei Y Church J Kutcher S. Long-term impact of a mental health literacy resource applied by regular classroom teachers in a Canadian school cohort. Child Adolesc Mental Health. (2022) 28:370–6. doi: 10.1111/camh.12597 36151716

[50] Kusaka S Yamaguchi S Foo JC Togo F Sasaki T. Mental health literacy programs for parents of adolescents: a systematic review. Front Psych. (2022) 13:816508. doi: 10.3389/fpsyt.2022.816508, PMID: 35586407 PMC9108239

[51] Ryan TG. Building mental health literacy within Ontario (Canada) health and physical education. Int Online J Educ Teach. (2020) 7:1252–64.

[52] Kutcher S McLuckie A. The child and youth advisory committee, Mental Health Commission of Canada. Evergreen: a child and youth mental health framework for Canada. Calgary, AB: Mental Health Commission of Canada (2010). Available at: https://www.mentalhealthcommission.ca/wp-content/uploads/drupal/Diversity_Evergreen_Framework_Summary_ENG_0_1.pdf (Accessed August 3, 2022).

